# The Interplay among Subunit Composition, Cardiolipin Content, and Aggregation State of Bovine Heart Cytochrome *c* Oxidase

**DOI:** 10.3390/cells9122588

**Published:** 2020-12-03

**Authors:** Erik Sedlák, Tibor Kožár, Andrey Musatov

**Affiliations:** 1 Center for Interdisciplinary Biosciences, P. J. Šafárik University, Jesenná 5, 040 01 Košice, Slovakia; tibor.kozar@upjs.sk; 2 Department of Biophysics, Institute of Experimental Physics, Watsonova 47, 040 01 Košice, Slovakia

**Keywords:** cytochrome *c* oxidase, aggregations state, cardiolipin, posttranslational modifications

## Abstract

Mitochondrial cytochrome *c* oxidase (CcO) is a multisubunit integral membrane complex consisting of 13 dissimilar subunits, as well as three to four tightly bound molecules of cardiolipin (CL). The monomeric unit of CcO is able to form a dimer and participate in the formation of supercomplexes in the inner mitochondrial membrane. The structural and functional integrity of the enzyme is crucially dependent on the full subunit complement and the presence of unperturbed bound CL. A direct consequence of subunit loss, CL removal, or its oxidative modification is the destabilization of the quaternary structure, loss of the activity, and the inability to dimerize. Thus, the intimate interplay between individual components of the complex is imperative for regulation of the CcO aggregation state. While it appears that the aggregation state of CcO might affect its conformational stability, the functional role of the aggregation remains unclear as both monomeric and dimeric forms of CcO seem to be fully active. Here, we review the current status of our knowledge with regard to the role of dimerization in the function and stability of CcO and factors, such as subunit composition, amphiphilic environment represented by phospholipids/detergents, and posttranslational modifications that play a role in the regulation of the CcO aggregation state.

## 1. Introduction

Bovine cytochrome *c* oxidase (CcO) is a protein–phospholipid complex, consisting of 13 nonidentical subunits with a combined molecular weight of ~205 kDa for the monomeric form of the enzyme [1]. The three largest subunits (I, II, and III) are encoded by mitochondrial genes. It should be noted that, throughout the manuscript, we use the subunit nomenclature according to Kadenbach [2,3]. Subunits I and II contain all of the redox-active centers: Heme *a*, heme *a*_3_, and Cu_B_ are located within the transmembrane region of subunit I; Cu_A_ is situated in the hydrophobic area of the intermembrane side of subunit II [4]. Four redox centers are involved in transport of electrons from ferrocytochrome *c* to Cu_A_, to cytochrome *a*, and finally to the binuclear center (cytochrome *a*_3_ and Cu_B_). Reduction of molecular oxygen in the binuclear center is coupled to the uptake of eight protons from the mitochondrial matrix. Four protons are translocated (pumped) to the intermembrane space, whereas the other four protons are used for the reduction of dioxygen to water. The 10 smaller subunits (IV, Va, Vb, VIa, VIb, VIc, VIIa, VIIb, VIIc, are VIII) are nuclear-encoded and are not present in bacterial CcOs, which are composed of 3–4 subunits [5]. Depending on the method of purification, the isolated enzyme sometimes contains an associated protein, NADH dehydrogenase (ubiquinone) 1 alpha subcomplex 4 (NDUFA4) [6,7].

However, such co-isolated proteins are not considered to be a constituent of the enzyme’s core [8] although it cannot be excluded that these subunits stabilize the monomeric form of CcO [4]. Previously, the role of individual CcO subunits was reviewed in detail by Kadenbach and Huttemann [3].

### 1.1. Specific Cardiolipin Interactions with CcO Surface

**Figure 1:**
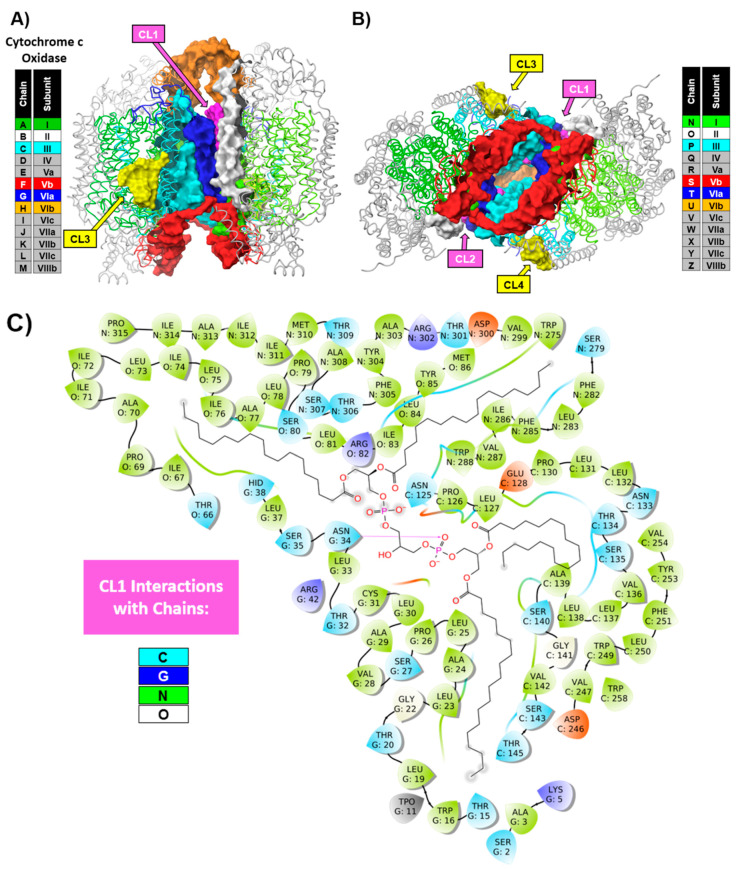
Colored ribbon representations and interface surface of cytochrome *c* oxidase (CcO; Protein Data Bank (PDB) structure 5B1A [12]). (**A**) Side and (**B**) bottom views. The color coding for the ribbons and subunit interface surfaces is as described in the inset boxes. The surface color coding matches the chains; the surfaces of cardiolipins CL1 and CL2 are colored in violet. In addition, the surfaces of CL3 and CL4 are highlighted in yellow. The other molecules present in the intersubunit cavity are not shown. (**C**) A schematic representation of the amino acids that interact with CL1 and CL2 residues present in the cavity between monomers as determined by the Maestro (Schrodinger Inc., New York, NY, USA) calculation of the interaction profiles. Note that, due to the symmetrical structure of CcO, the interaction of CL2 with chains of both monomeric CcOs is not depicted. The amino-acid color coding for the interaction profile is as follows: green—hydrophobic, light blue—polar; dark blue—charged (positive); orange—charged (negative); gray—unspecified residue or metal. Hydrogen bonds are highlighted with violet arrows.

The multisubunit enzyme complex spans the inner mitochondrial membrane and is in contact with an annulus of membrane phospholipids. In addition, there is a strong and specific requirement for nonannular, integral phospholipids, particularly cardiolipin (CL). According to our research, as well as the results of other groups, one can conclude that the fully functional form of CcO consists of the two high-affinity CL-binding sites. In our study, we identified CL-binding sites by photolabeling with arylazido-cardiolipin analogues and detecting labeled subunits by reversed-phase HPLC and HPLC/electrospray ionization mass spectrometry [9]. The high-affinity CL-binding sites were located near subunits VIIa, VIIc, and possibly VIII. The position of high-affinity CLs in each monomer, CL3 and CL4, were detected in the crystal structure [10] and are highlighted in Figure 1. The location of the binding site for the second high-affinity CL, which was not detected in the crystal structure, was identified between subunits VIIa and VIIc near the entrance to the D-channel. This position was later fully confirmed in the in-depth coarse-grained molecular simulation analysis [11] One more CL position in each monomer, highlighted in Figure 1 as CL1 and CL2, was located adjacent to subunit VIa. This CL had lower affinity and was only present in the 13-subunit form of CcO, playing a role in the dimerization of CcO.

### 1.2. Nonspecific Entropic Effect of Phospholipids on Dimerization of Membrane Proteins

The self-association of proteins to form dimers and higher-order oligomers is a common phenomenon in protein Biochemistryistry. The possible advantages provided by a homodimeric and oligomeric structures are increased stability and activity of the enzyme and an improved ability for regulation of its activity particularly through allosteric modulation. Specific dimerization is often a decisive factor in the regulation of proteins, ion channels, receptors, and transcription factors (for reviews, see [13,14,15]. However, in the case of integral membrane proteins, the advantages of dimer formation are less clear and, in many cases, not known. Although many critical bioenergetic and signaling events involve the transient or permanent assembly of proteins in membranes, the molecular interactions that affect protein association are often vague. The unusually frequent occurrence of dimeric structures for integral membrane proteins suggests that the lipid component of the membrane exerts entropic control over membrane protein assembly, playing a role similar to that of the water molecules producing the hydrophobic effect [16].

### 1.3. Specific Protein–Lipid and Protein–Protein Interactions in Dimerization of Membrane Proteins

Although entropically driven formation may be typical for transient complexes, the stability of more permanent dimeric and oligomeric structures is certainly modulated by enthalpically driven interactions intermediated by specific protein–protein and/or protein–lipid interactions. This is in accordance with so-called knob-into-hole packing that allows more efficient interaction between helices than between helices and lipids [17]. The presence of the GXXXG motif is thought to be a gauge of close packing and the tight, knob-into-hole packing, facilitated by the GXXXG motif (in which the glycines permit close approach of the helices), was found to be a general characteristic of helical bundle membrane proteins [18,19] Although a statistical survey of membrane protein sequences disclosed that such motifs are common in membrane proteins [20], our current knowledge suggests that the presence of a GXXXG motif alone is a weak predictor of membrane protein dimerization [21]. In fact, an analysis of helix dimers strongly indicates that transmembrane helix–helix interactions are modulated both by the sequence context and by the detergent and lipid environment [22]. The specific interactions of phospholipids with integral membrane proteins are intermediated by tightly bound phospholipids which can be (in most cases) identified in high-resolution crystal structures. These so-called nonannular phospholipids are usually bound in cavities and clefts of the integral membrane protein surface in an unusual position, i.e., the headgroup of the phospholipid is at the bilayer surface with acyl chains roughly in perpendicular position to the surface [23,24,25,26]. Phospholipids bound between subunits of integral membrane protein complexes seem to form a flexible interface between protein subunits, providing binding energy to stabilize ionic interactions and a flexible hydrophobic interface. Thus, this reduces the constraints on the types of amino-acid residues that can be present at the interface and increases the entropy of the interaction [16].

## 2. Role of Amphiphilic Environment in Aggregation State of Cytochrome *c* Oxidase

### 2.1. Evidence for Existence of Dimeric Structure of CcO

CcO is a typical example of a complex integral membrane protein for which the structural/functional role of the dimeric structure is not firmly established. Indeed, there is still no conclusive evidence whether the CcO “native” functional conformation is dimeric or monomeric within the mitochondrial inner membrane. For a long time, the general agreement was that CcO is dimeric in vivo. Several lines of evidence supported the idea that CcO may exist as a dimer in the inner mitochondrial membrane. For example, (i) the hydrodynamic size of the isolated CcO, as measured by gel filtration, was often interpreted as representing a dimeric complex [27,28,29,30]; (ii) dimeric CcO was detected by velocity and equilibrium sedimentation studies at neutral pH with a low concentration of nonionic detergent Triton X- 100 [31]; (iii) functional studies revealed the interaction of cytochrome *c* with the isolated enzyme [32]; (iv) in early studies, CcO was found to be certainly dimeric within both two-dimensional and three-dimensional crystalline arrays [33,34,35]. On the basis of these observations, it was suggested that the dimeric structure exists under physiological conditions. Moreover, in vitro, purified, monomeric CcO spontaneously dimerizes upon reconstitution into small unilamellar phospholipid vesicles, also suggesting that the natural mitochondrial environment leads to protein dimerization [36,37]. Thus, the dimerization of CcO occurring in the phospholipid bilayer, on one hand, and the existence of CcO as a monomer in mixed-detergent micelles, on the other hand, may be a result of the entropic role of phospholipids as suggested by Helms [16]. Noteworthy, it has been shown that, in another large family of intensively studied integral membrane proteins, G-protein-coupled receptors (GPCRs), an unfavorable hydrophobic mismatch, induced by lipid tail length and saturation, results in excessive dimerization, whereas lipid features such as anionic headgroups induce specific dimer interfaces via direct protein–lipid interactions [38]. Additionally, the observation that a higher degree of unsaturation in fatty acids causes a drastically reduced formation of compact receptor dimers due to facilitated lipid adhesion at the receptor surface supports the hypothesis of a lipid-entropy-driven transmembrane protein association [16,38].

### 2.2. Modulation of the Aggregation State of CcO by “Linear” Detergents

Despite many reports that CcO forms dimers, the dimerization of CcO has been for a long time an open question. A significant number of experimental studies have demonstrated that monomeric CcO is fully active. The observation that CcO exists in a dimeric form may, thus, be an incorrect consequence of a preparation artefact. Indeed, in spite of the fact that CcO is dimeric after reconstitution or crystallization, most preparations of isolated and detergent-solubilized CcO were either monomeric or a mixture of monomers and dimers. This may be due to different detergents and detergent concentrations favoring a different aggregation state of CcO. This observation is in agreement with earlier experimental findings that the dimerization thermodynamics of a transmembrane helix depends on the nature of detergent [39]. The discussion regarding the aggregation state of CcO dates back to the 1970s. For example, the review by Capaldi et al. [40] discussed the CcO aggregation state in relation to its electron-transfer activity. The paper cited previous publications which claimed the detergent- and source-dependent monomerization and dimerization of CcO [31,41,42,43]. Since then, numerous papers were published discussing self-association of CcO. The most comprehensive studies on self-association of isolated CcO and interaction of CcO with amphiphilic compounds were performed in the Robinson lab, in which CcO self-association was analyzed using an analytical ultracentrifugation technique in a variety of nonionic detergents, phospholipids, bile salts, and a mixture of surfactants [44,45,46,47,48,49,50]. Sedimentation velocity and sedimentation equilibrium represent a powerful tool for the quantitative analysis of macromolecules in solution in determining the molecular weight and aggregation state of integral membrane proteins and complexes. The most reliable approach for analyzing homogeneity, size, shape, and interactions of macromolecules is the sedimentation velocity technique. Sedimentation velocity data were analyzed using the van Holde–Weischet and the Stafford g*(s) methods [50,51]. However, interpretation of the measured sedimentation coefficients in terms of monomers or dimers is often quite difficult with detergent-solubilized proteins. This is evident from the published results since CcO has sedimentation coefficient values of 15–16S and 11–12S for the dimeric and monomeric forms, respectively, when solubilized in dodecylmaltoside, but 12–13S and 9–10S when solubilized in Triton X-100. Therefore, the monomeric dodecylmaltoside-solubilized enzyme has almost the same sedimentation coefficient as the dimeric Triton X-100-solubilized complex. For this reason, once homogeneity was established, the sedimentation equilibrium measurements were performed in order to verify the interpretation of the sedimentation velocity data and to determine the exact protein molecular weight. Collected sedimentation equilibrium data were analyzed as suggested by Tanford et al. [52] to correct for the altered hydrodynamic size and effective partial specific volume due to bound detergents and phospholipids. Evaluation of the detergent-solubilized protein molecular weight has also been done via a combination of the global fitting of sedimentation velocity data using the finite-element analysis method of Demeler and Saber [53] with the density variation method of Reynolds and Tanford [54]. Use of this program was described previously [45,46,47,48,49,50,51,52,53,54,55], and a new version is available together with tutorial examples on the web at http://ultrascan.aucsolutions.com/. It should be noted that a dependence of the aggregation state of CcO on the subunit composition was also monitored by reversed-phase HPLC. In a single detergent, CcO was never completely dimeric but was usually a mixture of monomers and dimers. Dodecylmaltoside, undecylmaltoside, and Triton X-100 were the most effective detergents for solubilizing CcO, but each of these produced mixtures of monomeric and dimeric enzymes at low concentrations (~2 mM) and completely monomeric enzyme at high concentrations (~20 mM). The maximum percentage of CcO dimers, 60–85%, occurred when CcO was solubilized by dodecylmaltoside. Increasing the pH produced a higher percentage of monomers. Decylmaltoside, octaethyleneglycolmonododecyl ether (C12E8), Tween-20, sodium cholate, sodium deoxycholate, CHAPS, and CHAPSO were all ineffective solubilizing detergents and CcO remained aggregated.

### 2.3. Dimerization of CcO Induced by “Planar” Bile Salt Detergents

In addition to a thorough analysis of CcO sedimentation behavior in different single surfactants, it was discovered that bile salts induce CcO dimerization [47]. More precisely, bovine heart CcO solubilized by either nonionic detergents, such as dodecylmaltoside, decylmaltoside, and Triton X-100, or phospholipids completely dimerizes upon the addition of bile salts, e.g., sodium cholate, sodium deoxycholate, or CHAPS. In each case, complete CcO dimerization was verified by sedimentation velocity and sedimentation equilibrium after correction for bound detergent and/or phospholipid. It was concluded that (i) CcO is dimeric when it is reconstituted into phospholipid vesicles in the presence of cholate, the procedure normally used to quantify proton pumping activity, (ii) detergent-solubilized, dimeric CcO can be routinely prepared by the addition of bile salts under solution conditions, (iii) cholate-induced generated dimers are stable even at relatively high pH and low enzyme concentration, (iv) dissociation into monomers is reversible, and (v) nuclear-encoded subunits VIa and VIb are required for dimerization. Two explanations for the ability of bile salt to induce CcO dimerization seem possible [47]. At first, the hypothesis of “surfactant rearrangement” assumes that sodium cholate triggers the rearrangement of CcO-bound detergents or phospholipids, permitting the necessary monomer–monomer contacts. The second and probably more “biological” explanation assumes bile salt binding at CcO specific sites induces conformational changes followed by dimerization. Bovine heart CcO contains up to 10 binding sites for ADP/ATP which could be occupied during the extraction procedure by sodium cholate [56]. Therefore, it is tempting to speculate that perturbations of the oligomeric state of CcO are regulated by nucleotides. This hypothesis was recently supported by Ramzan et al. [8]. The authors showed that, in mitochondria, CcO dimerization/monomerization transitions are regulated by nucleotides via reversible phosphorylation. Moreover, according to the X-ray crystallographic structure of CcO, it was also proposed that the dimeric structure can be stabilized by ATP or cholesterol, i.e., by physiological compounds with a planar structure, with the case of cholesterol being structurally similar to cholate [4]. On the other hand, the observation that cholesterol may modulate the dynamic properties of another family of integral membrane proteins, GPCRs, with a direct effect on equilibrium between monomers and dimers suggests a more general effect on modulation of monomer/dimer equilibrium of integral membrane proteins through the composition of membrane components. This would be the case for a component with a planar structure such as for cholesterol [38,57,58,59].

Last but not least, it should also be noted that individual preparations of the enzyme behave somewhat differently. For example, isolation methods utilizing sodium cholate may generate rather than stabilize dimeric CcO (note: CcO used for structure determination was purified in the presence of sodium cholate, except in a very recent study by Shinzawa-Itoh et al. [4]). On the other hand, isolation of mitochondrial CcO using high concentrations of Triton X-100 resulted in homogeneous and monomeric CcO [47]. Even when Triton X-100 was exchanged for dodecylmaltoside during the last purification step, we were unable to induce dimerization of CcO via the addition of bile salts [47]. Presumably, this is due to the very high concentration of Triton X-100 in such preparations. In fact, it was estimated that CcO binds about 180 ± 30 mol of Triton X-100/mol of CcO [44]. Mostly monomeric CcO was observed by blue native PAGE of dodecylmaltoside-solubilized mitochondrial complexes [60,61].

## 3. Role of Interface Subunits and Cardiolipin in Function and Stability of Cytochrome *c* Oxidase Dimeric Form

**Figure 2:**
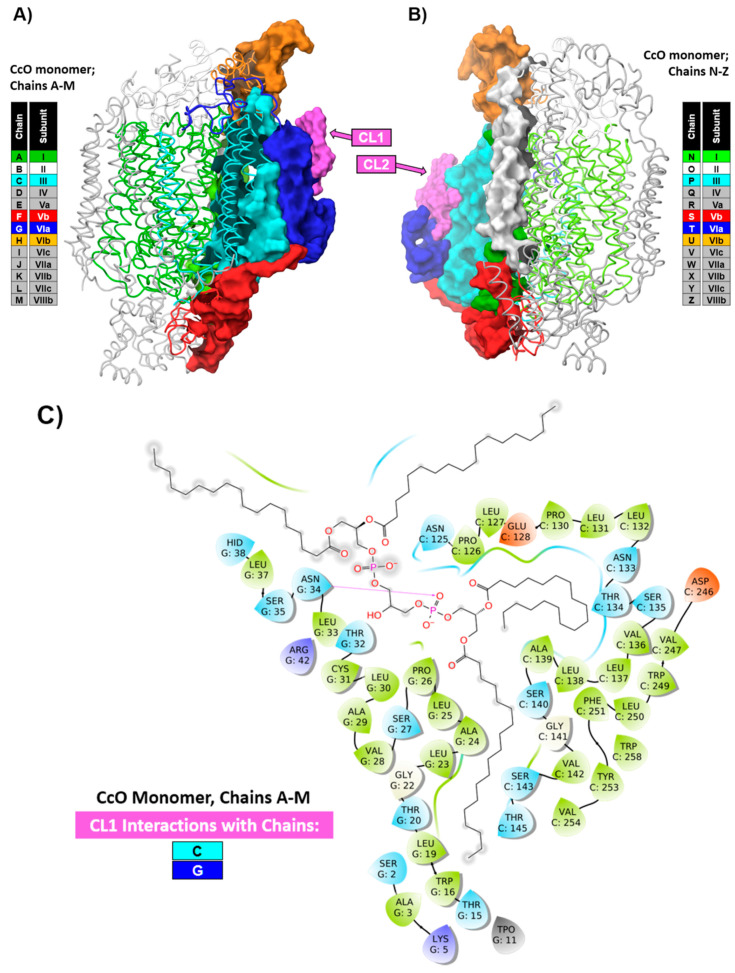
Colored ribbon representations of cytochrome *c* oxidase (PDB structure 5B1A [12]) split into monomers (**A**,**B**). The color coding for the ribbons and subunit interface surfaces is as described in the inset boxes. The surface color coding matches the chains; the surfaces of cardiolipins CL1 and CL2 are colored in violet. The other molecules present in the intersubunit cavity are not shown. (**C**) A schematic representation of the amino acids that interact with CL1 bound to A–M chains as determined by the Maestro (Schrodinger Inc., New York, NY, USA) calculation of the interaction profiles. Due to the symmetrical structure of CcO, the interactions of CL2 with N–Z chains are not shown. The number of interactions significantly decreased in comparison to the dimer structure of CcO. See Figure 1 for color coding of the amino acids shown in interaction profiles.

In the preceding section, we discussed the independent role of the surrounding amphiphilic environment in the inherent structural properties of CcO. A reasonable assumption regarding the proper formation of a CcO dimeric state, in addition to a proper amphiphilic environment, is the intact monomeric structure of CcO, i.e., the presence of all 13 subunits including at least one CL molecule per monomer localized in the cavity formed in the intermonomeric interface of dimeric CcO (Figure 1). Overall, according to Lee et al. [62], the dimer is stabilized by four subunit–subunit contacts, namely, subunit Vb–subunit Vb, subunit I–subunit VIa, subunit VIa–subunit I, and subunit VIb–subunit VIb. In fact, the first X-ray crystallographic analysis of the three-dimensional (3D) structure indicated that subunits VIa and VIb form the major contact between monomers with a critical role in the stabilization of the dimeric structure of CcO [35]. This offers an alternative explanation why CcO, purified using a high concentration of TX-100, is in monomeric form. In the presence of a high detergent concentration during isolation and purification procedures, a partial or complete dissociation of subunits VIa and VIb may occur, resulting in the inability of the CcO monomers to dimerize. Our previous work with the delipidated form of CcO [63,64], the photolabeling studies of CcO with CL analogues [9], the crystal structure analysis [10,26], and the coarse grain molecular dynamics simulation [11] together suggest a model in which there is a tight interaction of 2–3 molecules of CL with the CcO monomer. This interaction would have distinct structural and functional roles. Here, we provide an analysis of the interaction between CL and CcO in the intermonomeric interface. The calculation of the interaction profiles of CL with CcO clearly indicate numerous interactions of CL1 (and CL2) with the subunits III and VIa within the CcO monomer (Figure 2).

### 3.1. Role of Unspecific Phospholipids in Stabilization of Dimeric CcO

**Figure 3:**
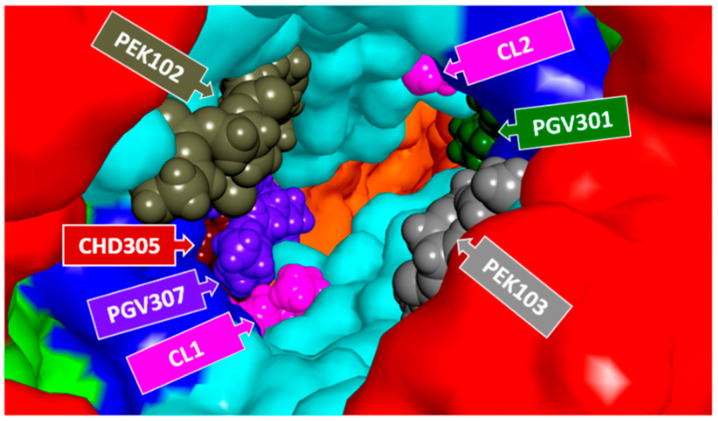
Visualization of hydrophobic molecules bound in the intermonomer cavity of CcO (PDB structure 5B1A [12]) probably as a part of crystallization solvent. CPK visualization of PGV ((1*R*)-2-{[{[(2*S*)-2,3-dihydroxypropyl]oxy}(hydroxy)phosphoryl]oxy}-1-[(palmitoyloxy)methyl]ethyl (11*E*)-octadec-11-enoate), PEK ((1*S*)-2-{[(2-aminoethoxy)(hydroxy)phosphoryl]oxy}-1-[(stearoyloxy)methyl]ethyl (5*E*,8*E*,11*E*,14*E*)-icosa-5,8, 11,14-tetraenoate), and CHD (cholic acid) present in the cavity of cytochrome *c* oxidase.

This analysis is in full agreement with our published findings in which it was shown that enzymatic hydrolysis of tightly bound CL molecules is accompanied by dissociation of subunits VIa and VIb [63]. The analogous analysis of bound CL molecules with CcO in dimeric form shows numerous hydrophobic, charge–charge, and hydrogen bond intermonomeric interactions within CL1 (and CL2) with subunits III and VIa on one monomer and subunits I and II on the second monomer (Figure 1). The many bridging interactions stabilize the dimeric structure of CcO and support the existence of a stable dimeric form of CcO. Advanced mass spectrometry methods in which the oligomeric state of CcO was investigated strongly support a stabilization role for lipids in the dimeric form of CcO [1]. The authors determined that the lipids associated with dimeric CcO constitute a total mass of 25 kDa. Their analysis identified five lipid classes (phosphatidylserine (PS), phosphatidylethanolamine (PE), phosphatidylinositol (PI), phosphatidylglycerol (PG), and phosphatidylglycerolphosphate (PGP)), with several isomers and a number of CsL. This lipid analysis also revealed three lipid classes previously unidentified with bovine CcO, namely, PI, PS, and PGP. As the authors pointed out, these lipids are only minor constituents of the mitochondrial inner membrane [65] and are, therefore, likely sequestered from exchange with bulk lipids in the membrane (PE and PC) by virtue of their specific binding in the dimer interface. These phospholipids together with previously identified CLs in the dimeric interface form a “lipidic plug” that seals the dimeric space with possible stabilization and functional role (Figure 5 in Liko et al. [1]). An analysis of the crystal structure of CcO (Protein Data Bank (PDB) ID: 5B1A) also suggests the existence of a lipidic plug between CcO monomers. The dimeric interface of this CcO structure consists of, in addition to tightly bound CL molecules, hydrophobic molecules that were apparently a part of the crystallization mixture (Figure 3). The stabilization role of the hydrophobic dimeric interface is likely achieved predominantly by the presence of CL molecules due to their ability to function as a type of “hydrophobic glue”. This is a result of protein–CL interactions, as well as their flexibility and unusual bidentate structure, all of which allow a link of relatively distant (intra and/or inter) sites in the CcO dimer [1,66]. The lipidic plug likely plays a critical role in preventing leakage of the inner mitochondrial membrane, a crucial feature in the production of ATP by complex V of the respiratory chain.

### 3.2. Relationship between Specific CL Binding/Dimerization and Proton Pumping of CcO

On the basis of the above structural analysis of CcO, one can conclude that the functional form of CcO is a dimer in the phospholipid bilayer. However, is it? Do we have solid data supporting the structural analysis? In fact, a commonly used argument that the proton pumping activity of CcO is observed only in the dimeric form of CcO is not so robust. Most of a preparation of CcO dimerizes after incorporation into liposomes, a critical caveat given that liposomes are routinely used for proton pumping measurements. Previously, an attempt was made to use CL-free monomeric enzyme [49]. The proton pumping activity of CL-depleted CcO was completely abolished when all of the bound CLs were removed by the procedure described by Sedlak and Robinson [63]. In addition, CL-free CcO reconstituted to phospholipid vesicles exhibited low respiratory control ratios and a random orientation in liposomes. Moreover, the resulting monomeric enzyme lost subunits VIa and VIb, which are required for dimerization. Therefore, it is unclear whether monomers are incapable of proton pumping activity or whether removal of CL alone is sufficient to inhibit proton translocation either through an indirect destabilization effect of removed CL on CcO conformation or through a direct effect of CL assuming its role as a proton antenna due to localization of its headgroup adjacent to the entrance to the D-channel [9]. The indirect effect on the proton pumping ability of CcO, appears to be more likely. In fact, enzymatic hydrolysis used to remove CL from CcO [63] hydrolyzes all phospholipids. In such a case, the lipidic plug in the dimeric interface would be removed, the delipidated dimeric CcO (if existed) would miss stabilizing subunits VIa and VIb, and a proton would leak through the emptied dimer interface. This may provide an explanation for the undetected proton pumping activity of delipidated CcO, as well as the extended proton leakage, observed in the case of the CcO delipidated form [49]. It is also possible that the removal of CL will irreversibly affect CcO conformation, preventing its proton pumping activity. In accordance with this conclusion, Berg et al. [67] very recently showed that minor structural changes at the orifice of the D pathway, at a distance ~40 Å from the proton gate of CcO, may affect the proton pumping of the enzyme.

### 3.3. Evidences for a Monomeric Electron-Transfer Competing Form of CcO

Does this mean that functional CcO exists as monomer? The existence of a functional monomeric form of CcO is supported by the results of several recent studies [4,8,68,69]. This includes bovine heart CcO incorporated into bicelles, bilayered long- and short-chain phospholipid assemblies, characterized by absorption spectroscopy, dynamic light scattering, atomic force microscopy, small-angle neutron scattering, sedimentation velocity, and differential scanning calorimetry [68,69]. CcO in bicelles was fully reducible by artificial donors of electrons, exhibited a “normal” reaction with external ligands, and was fully active. Sedimentation velocity analysis, small-angle neutron scattering, and temperature-induced denaturation indicated that the enzyme in bicelles is monomeric. The authors concluded that CcO in bicelles maintains its structural and functional integrity. Shinzawa-Itoh and colleagues [4], using amphipol, prepared stabilized monomeric and dimeric bovine CcO to show that the monomer had higher activity than the dimer. In addition, using a newly synthesized detergent, they determined the oxidized and reduced structures of monomer with a resolution of ~1.9 Å. The structural changes observed for monomeric CcO were essentially the same as those observed previously for dimeric CcO. The authors, thus, concluded that (i) observed structural changes are not dependent on crystal packing and (ii) the difference in activity between monomeric and dimeric CcO does not depend on the difference in redox-dependent structural changes between monomeric and dimeric CcO. Moreover, structural analysis revealed that a hydrogen bond network of water molecules was formed at the entry surface of the proton transfer pathway, termed the K-pathway, in monomeric CcO, whereas this network was altered in dimeric CcO. According to these findings, the authors proposed that the monomer is the activated form, whereas the dimer can be regarded as a physiological standby form in the mitochondrial membrane [4]. The work of Ramzan et al. [8] partially supports this finding. The authors studied two forms of CcO: (i) a dimeric structure, isolated using cholate as detergent, and (ii) a monomeric enzyme, isolated with nonionic detergents. Their data indicate a continuous in vivo transition between CcO monomers and dimers via reversible phosphorylation. Interestingly, the monomeric CcO was more active, in accordance with the Shinzawa et al. [4] report, while dimeric CcO exhibited an “allosteric ATP inhibition”. This inhibition affects respiration at high cellular ATP/ADP ratios and could prevent oxygen radical formation, a negative effector on the generation of mitochondrial diseases [8].

### 3.4. Fast Labile Monomer versus Tunable Stable Dimer of CcO

On the basis of our discussion, we can conclude that both dimeric and monomeric forms of CcO are functionally competent. While it seems that the monomeric form of CcO is more active than the CcO dimer [4,8], in our opinion, there are two critical advantages of the dimeric enzyme over the monomeric form: (i) the higher stability of the CcO homodimer and (ii) the ability to finely tune the homodimer CcO activity. The first conclusion is based on two studies of Robinson’s group in which the stability of different forms of CcO was tested by elevated hydrostatic pressure and increased temperature [48,70]. In the first study, the authors showed a significantly different quaternary stability of monomeric and dimeric detergent-solubilized CcO exposed to hydrostatic pressure. Dimeric CcO could withstand 2–3 kbar of hydrostatic pressure without loss of subunits or enzymatic activity, while monomeric enzyme exhibited only ~40% of electron-transport activity and a partial or total loss of subunits VIa, VIb, III, or VIIa [48]. In the second study, thermally induced transitions of monomeric and dimeric forms of bovine CcO were studied by differential scanning calorimetry and circular dichroism. Kinetic analysis of differential scanning calorimetry experiments enabled us to show that the kinetic stability of the enzyme significantly increases upon dimerization induced by sodium cholate CcO [70]. Interestingly, the first step of thermal denaturation involves dissociation of the same subunits III, VIa, VIb, and VIIa at the elevated hydrostatic pressure. We showed that the dimer interface consisting of the subunits III, VIa, VIb, and VIIa is the most fragile part of CcO, and these subunits tend to dissociate from the core of the enzyme upon external perturbations such as elevated pressure [48], increased temperature [70], increased concentration of denaturants [71], and oxidative stress [72,73,74,75,76]. Dimerization of CcO aided by CL molecules may be a way to protect the most fragile part of the enzyme against external effects. The second statement, regarding a better ability to tune the activity of the dimeric form, is based on numerous experimental results published by Kadenbach’s group [77,78,79,80,81]. Moreover, Ramzan et al. [82] very recently reviewed the mechanism that includes the loss of allosteric ATP inhibition of CcO involving the formation of reactive oxygen species.

## 4. Posttranslational Modifications in Cytochrome *c* Oxidase

To date, 18 phosphorylation sites have been identified in the seven different mammalian CcOs. However, according to the consensus sequences for protein kinases [83], up to 53 potential phosphorylation sites may occur in the bovine CcO when one considers just serine and threonine sites [84]. In addition, a total of 14 acetylated sites were recently identified in the 13 CcO subunits [1]. The effect of reversible phosphorylation of certain subunits on CcO electron-transfer activity was previously discussed [3]. The relationship between phosphorylation and association state of CcO was demonstrated only recently [8]. According to the authors, reversible dimerization of CcO in mitochondria is induced by cAMP-induced activation of a protein kinase, which phosphorylates subunit I on the intermembrane side and is accompanied by the “allosteric ATP inhibition”. Small concentrations of calcium activate a protein phosphatase which dephosphorylates this site and monomerizes the CcO dimer. This is the only demonstrated example of a connection between the oligomeric state and posttranslational modification in CcO. Liko et al. [1] compared their results with previous studies and found that the majority of acetylated residues are conserved across species (human, mouse, and rat). From the crystal structure of the CcO dimer (PDB ID: 2OCC), it follows that phosphorylation occurs primarily on the matrix side of the soluble subunits, and acetylated lysine residues are more widely distributed and often in proximity to phosphorylation sites. Thus, although acetylation of lysine residues is accompanied by a loss of positive charge and, thus, may affect protein–protein or protein–lipid interactions, the wide distribution of acetylated amino acids over the structure suggest that, if such an effect does exist, it is triggered indirectly. As the authors noted, the changes in the interactions that occur between proteins that have undergone posttranslational modification and associated lipids is a relatively new concept that may have major implications in the organization of membrane–protein complexes.

## 5. Cytochrome *c* Oxidase in Supercomplexes of the Respiratory Chain

The aggregation state of CcO has lately received a great deal of attention. It started with structural analyses of the mitochondrial respiratory supercomplexes (respirasomes) which demonstrated association of monomeric CcO with complex I and complex III. The term respiratory supercomplex is a synonym for the association of individual mitochondrial respiratory chain complexes. Although there were previous experimental indications of steady interactions between individual respiratory complexes (for a review, see [85]), the first work describing supercomplexes within bovine heart mitochondria was published by Schägger and Pfeiffer [86]. Mitochondria were treated under mild solubilizing conditions with blue native PAGE used for detection. Almost all complex I was found in association with dimeric complex III and up to four copies of complex IV (SCI1III2IV1–4). In subsequent research, the most predominant isolated and analyzed supercomplex form was SCI1III2IV1 [87,88,89,90,91]. For example, single-particle cryo-electron microscopy at 5.4 Å resolution described the intersupercomplex interaction between the individual respiratory chain proteins [91]. The specific protein–protein contacts within the bovine SCI1III2IV1 supercomplex were also reported at 9 Å resolution [92]. Despite intensive studies of structural properties of supercomplexes, the kinetic advantage of bringing complexes III and IV into proximity within the membrane to form supercomplexes was demonstrated only recently [93]. The findings provided novel evidence that a functionally relevant monomeric CcO exists in vivo. Indeed, almost all structural data of supercomplexes demonstrated the presence of monomeric CcO. It is noted that one possibility among the models for respiratory strings was based on the dimerization of CcO, connecting individual supercomplexes SCI1III2IV1 [94,95]. However, this is very unlikely since it may induce unnatural membrane curvature [96]. Similarly, fitting the CcO dimer into the respiratory megacomplex is questionable due to clashes with complexes I and III [97]. A strong argument supporting the monomeric structure of CcO was already discussed by Shinzawa-Itoh et al. [4]. On the other hand, studies of the purified mammalian and yeast respiratory chain supercomplexes by single-particle cryo-electron microscopy (cryo-EM) [89,98] and cryo-electron tomography [99] suggest that SCI1III2IV1 might be a consequence of the presence of an excess of CLs localized between transmembrane domains of individual complexes. The function of CL in such a case would be stabilization/gluing together of “less natural” supercomplex SCI1III2IV1. It is noted that, SCI1III2IV1 differs from supercomplex SCIII2IV2 in that SCIII2IV2 is structurally distinct consisting of “natural” dimeric forms of complexes III and IV. Consistent with these observations, a great excess of CL was tightly associated with and integrated into the structure of the individual purified complexes in the case of supercomplex SCI1III2IV1. Here, about 200 CL molecules were estimated to be present per supercomplex [89], in comparison with approximately 50 CL molecules in the *Saccharomyces cerevisiae* III2IV2 supercomplex. In conclusion, on the basis of observations that CcO is active in all three aggregation states, it is hypothesized that the enzyme in vivo can exist as a dimer and monomer within the supercomplexes.

## 6. Conclusions

Despite significant progress in our understanding of the function of mitochondrial CcO and the determination of several crystal structures of CcO at high resolution, the question of whether the functional form of CcO is a monomer or dimer remains open. The entropic effect of the lipid component of the membrane suggests that integral membrane dimerization is a consequence of membrane thermodynamics. This follows from the fact that both of these forms are capable of electron transfer. Moreover, recent findings suggest that the monomer CcO is more active than the dimer, although the issue whether the monomer form is an efficient proton pump remains unresolved. Alternatively, the presence of a lipidic plug and CL in the dimeric interface with a stabilization role suggests a stabilization and/or functional role of dimerization for CcO. We believe that, according to the up-to-date findings, one can conclude that the dimer form is more stable and more tightly regulated than the monomer form of CcO. Surprisingly, despite the critical physiological importance of CcO, our understanding of the regulation (if ever existing) of the enzyme oligomeric state by posttranslational modifications and composition of the phospholipid bilayer can be still considered to be in its infancy.
